# Longitudinal Visual Biomarkers in Dominant Optic Atrophy: A Systematic Review and Meta‐Analysis

**DOI:** 10.1111/ceo.14543

**Published:** 2025-05-07

**Authors:** Christopher A. Ovens, John R. Grigg, Clare L. Fraser

**Affiliations:** ^1^ Save Sight Institute, Faculty of Medicine and Health The University of Sydney, Sydney Eye Hospital Campus Sydney Australia; ^2^ Eye Genetics Research Unit, The Children's Hospital at Westmead, Save Sight Institute, Children's Medical Research Institute The University of Sydney Sydney Australia; ^3^ Sydney Eye Hospital Sydney Australia

**Keywords:** autosomal dominant optic atrophy, biomarkers, review

## Abstract

**Background:**

Dominant Optic Atrophy (DOA) causes slowly progressive visual decline usually beginning in childhood. As new therapies come to clinical trial, the choice of biomarkers to be used as clinical trial endpoints has become a critical question to be addressed.

**Methods:**

We undertook a systematic review and meta‐analysis of studies reporting longitudinal data of any biomarker in DOA patients.

**Results:**

In total, seven studies were included in the systematic review, and four presented paired results compatible with meta‐analysis. Visual acuity was the only biomarker found with reported longitudinal data. Of the included studies in the meta‐analysis, the rate of yearly visual acuity decline (0.022 LogMAR/year., 95% CI: −0.008 to 0.052) was not significantly different from zero (*Z* = 1.4, *p* = 0.155).

**Conclusion:**

Quantifying this slow rate of visual decline has implications for future study design and suggests that further natural history studies examining alternative biomarkers in DOA are warranted.

## 1 Introduction

Dominant Optic Atrophy (DOA) is a progressive disease of retinal ganglion cells leading to visual loss [1]. The clinical presentation of DOA includes: an insidious onset of bilateral visual loss most commonly in the first two decades of life; generalised dyschromatopsia or pure tritanopia; cecocentral or paracentral visual field defects; and classical temporal or diffuse optic disc pallor [2, 3, 4]. It has an estimated prevalence of 1 in 30 000, with penetrance of at least 70% [5]. OPA1 is a dynamin‐related protein of the large GTPase superfamily that locates to the inner mitochondrial membrane and is integral for mitochondrial structure and function [6]. Mutations in the *OPA1* gene account for approximately 75% of DOA cases, typically via a haploinsufficiency mechanism causing reduced OPA1 protein function [5]. However, significant phenotypic and genotypic heterogeneity in DOA patients is increasingly being described.

The development of lenadogene nolparvovec (rAAV2/2‐ND4) for Leber Hereditary Optic Neuropathy patients [7] heralds the emergence of genetically targeted therapies for optic neuropathies. There is currently no disease‐modifying treatment for DOA, although idebenone has been shown to improve visual outcomes [8]. The development of novel therapies for DOA, such as the antisense oligonucleotides PYC‐001 [9] and STK‐002 [3], will require clinical trials to assess their safety and efficacy. Several features of DOA make it a difficult disease to study in the clinical trial setting. First, the genetic heterogeneity among DOA patients leads to significant inter‐ and intrafamilial variation, which is likely to influence treatment response. Second, as a rare disease [10], there are difficulties in optimising study power and minimising type II errors. Finally, clinical progression of the disease tends to occur slowly and, therefore, may be difficult to detect. Thus, we must have a thorough understanding of both the genetic basis of the disease as well as the natural history of progression to enable future clinical trials to detect meaningful responses to these emerging DOA therapies.

This systematic review sets out to establish natural history data for available biomarkers in DOA patients. We aim to inform future study design for emerging therapies in DOA, as well as improve prognostication when counselling patients regarding the expected disease course.

## 2 Methods

A protocol was developed by the study authors and was registered on the PROSPERO database (CRD42024548499). This was developed in accordance with the updated PRISMA guidelines [11]. We included all studies with longitudinal data of genetically‐proven DOA patients, whether retrospective or prospective. All biomarkers with longitudinal data were included, regardless of the specific reporting methods for each biomarker. Cross‐sectional studies without longitudinal data, or where longitudinal data was unable to be extracted, were excluded. Inclusion and exclusion criteria are outlined in Table 1.

**TABLE 1:** Study inclusion and exclusion criteria.

Inclusion criteria: – Studies of genetically confirmed cases of DOA/DOA+ (*OPA1* only) – Longitudinal quantitative data reported (interventional or natural history studies), with follow‐up ≥ 12 months – 1 or more clinical biomarker used (e.g., visual acuity, perimetry, optical coherence tomography (OCT), electrophysiology etc.) – Limited to English language and human subjects	Exclusion criteria: – No confirmed genetic diagnosis for DOA, or different gene – Patients in the intervention arm of interventional studies – Cross‐sectional studies lacking longitudinal data, or reports where biomarker data is not readily available. – Case reports – Unpublished or abstract materials

Systematic literature search was performed using Medline Ovid, EMBASE Ovid, Cochrane CENTRAL, ClinicalTrials.gov and the World Health Organisation International Clinical Trials Registry Platform, all from inception. Index (MeSH) terms and free text keywords were used to search the following concepts: (1) A diagnosis of Dominant Optic Atrophy, (2) any use of biomarkers, (3) human studies (see Appendix A for full search strategy). This was adapted depending on the database used. All databases were last searched on May 1, 2024. Manual reference list search was also performed to identify any papers potentially missed in the initial search. Grey literature including conference abstracts, presentations, unpublished articles or research theses was not included.

Initial results of the literature search were loaded into EndNote 21 (Thomson Reuters) and duplicates removed. Two authors (C.O., C.F.) performed an independent abstract review using Covidence systematic review software (Veritas Health Innovation, Melbourne, Australia) to exclude irrelevant studies. Abstracts with insufficient information to apply our eligibility criteria were carried forward to the full‐text screening stage. A full‐text review was then performed by two authors (C.O., C.F.) independently against the inclusion and exclusion criteria, and reasons for exclusion were documented. Any disagreement was resolved with the assistance of a third reviewer (J.G.), and consensus was reached.

Data extraction was performed by two authors independently using standardised forms. Extracted data included: (1) study characteristics (publication year, country or region, study design, population description and size, biomarkers used); (2) patient characteristics (genetic diagnosis and phenotype, sample size) and (3) biomarker data (biomarkers with longitudinal data, biomarkers without longitudinal data, whether primary or secondary endpoint and whether this was met [if used in clinical trial], baseline and follow up mean values and measure of spread, mean follow‐up, mean rates and range of change over time, and any other biomarker data given). If not directly reported, rate of change data was calculated post hoc from reported paired data when available. Biomarker characteristics (test–retest variability, correlation with other biomarkers, interocular variability) were also noted where reported. These were reported in table format and discussed in a narrative summary. A meta‐analysis was performed using data from studies where paired rates of change were reported or able to be extracted post hoc. The analysis was carried out using the effect‐size as the outcome measure using a random‐effects model. The *Q* test statistic and *I* ^2^ statistic was used to assess heterogeneity.

Risk of bias was assessed based on study design. For prospective case–control, cohort, or randomised control trials (RCTs), the Newcastle‐Ottawa Scale [12] was used. For case series and natural history studies without a comparator group, the JBI Critical Appraisal Checklist for Case Series [13] was used. This was addressed in narrative form.

## 3 Results

**FIGURE 1:**
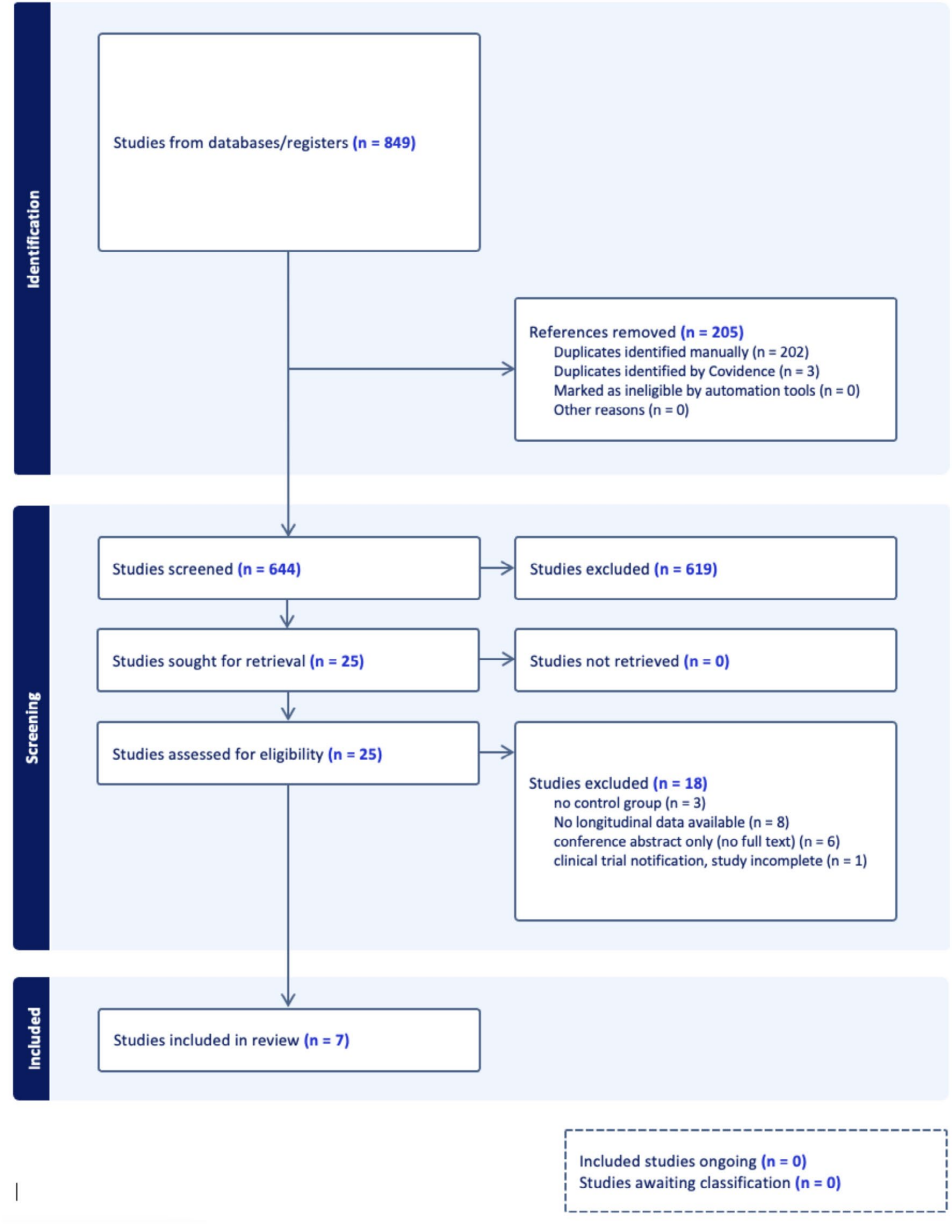
PRISMA flow chart of systematic literature search and screening.

The initial search strategy yielded 849 results. After duplicates were removed, 644 papers underwent abstract screening. The authors selected 25 papers as either meeting eligibility criteria or as inconclusive to progress to full‐text review. Independent full‐text review found seven articles meeting our eligibility criteria, with 18 studies excluded at this step. This is summarised in Figure 1, including reasons for exclusion at the full‐text stage.

The majority of studies were cross‐sectional in nature, lacking any paired longitudinal data. Of those studies included, only one was an interventional study reporting the effect of idebenone in DOA [8]. The remaining studies were natural history studies following DOA patients over time (Table 2).

**TABLE 2:** Summary of longitudinal data available from included studies.

Study (Location)	*n*	Biomarkers used	How reported?	Result	Mean follow‐up
Kjer 1996 [14] (Denmark)	30	VA	Proportions (Definitions of progression not stated) Post hoc mean lost LogMAR/year. (± SD)	10/30 stable 20/30 progressed 0.024 (± 0.030)	14 years (range 1–38 years)
Puomila 2005 [15] (Finland)	20	VA	Proportions (> 2 lines = significant change) Post hoc mean lost LogMAR/year. (± SD)	10/20 lost > 2 lines 10/20 lost < 2 lines 0.017 (± 0.020)	13 years (range 1–37 years)
Cohn 2008 [16] (Australia)	69	VA Optic disc appearance	Proportions: Cohort BCVA BL vs. LFU: Fundoscopic features	7/69 gained > two lines, 43/69 unchanged, 19/69 lost ≥ two lines BL: mean 6/18, median 6/24, range 6/4.8‐HM LFU: mean 6/18, median 6/36, range 6/4‐HM No change	9.6 years (SD ± 7.9)
Yu‐Wai‐Man 2010 [4] (Northern England)	43	VA	Cohort mean (± SD) LogMAR BCVA BL vs. LFU Rate of change LogMAR/year. (± SD)	BL: 0.68 (± 0.53) LFU: 1.06 (± 0.67) 0.032 (± 0.045)	18 years (SD ± 14.7)
Yu‐Wai‐Man 2011 [17] (USA)	24	VA	Cohort mean (± SD) LogMAR BCVA BL vs. LFU Rate of change LogMAR/year. (± SD)	BL: 0.45 (95% CI: 20/48–20/66) LFU: 0.66 (95% CI: 20/68–20/124) 0.070 (± 0.098)	8.5 years (SD ± 9.0)
Romagnoli 2020 [8] (Italy)	37	VA	Cohort median BCVA BL vs. LFU:	BL: 0.52 (Q1–Q3, 0.2–0.9) LFU: 0.52 (Q1–Q3, 0.3–1.0)	3.4 years (SD ± 2.5)
Arruti 2023 [18] (Spain)	11	VA	Cohort mean BCVA BL vs. LFU:	BL: Right: 0.40, Left: 0.44 LFU: Right: 0.40, Left: 0.44	unknown (study run over 4 years)

The first reported longitudinal study of DOA patients with supporting genetic results was published in 1996 by Kjer et al. [14]. These patients all showed genomic linkage to the telomeric region of chromosome 3q, which was subsequently found to be the location for *OPA1*. Thirty of the 62 Danish patients reported had retrospective longitudinal data available. Mean observation time was 14 years (range 1–38 years). 10/30 patients showed no progression. The remaining 20 showed visual deterioration, although what was considered ‘deterioration’ was not defined. It was also noted that the rates of vision loss were variable, with five patients showing more rapid decline. The authors noted variation within and between families. Although not directly reported, the rate of visual change was calculated post hoc according to the published figure. The rate of change in LogMAR visual acuity was calculated as 0.024 (± 0.030) LogMAR/year. In this cohort, refractive state, colour vision, Goldmann visual field, and optic disc assessment were also reported, but no longitudinal data was available.

A follow‐up study in 2005 from Puomila et al. [15] reported the ophthalmic and genetic findings in 14 Finnish families. Of the 54 included patients in known DOA families, 33 were affected. Eight patients had normal visual acuity at the time of first screening and where visual loss was present it was typically symmetrical. Of these 33 patients, 20 were followed for over 6 years with results of the better eye reported. Half had visual deterioration ≥ 2 lines in both eyes, while half remained stable. Post hoc analysis found the rate of change in LogMAR visual acuity was calculated as 0.017 (± 0.020) LogMAR/year. Visual field, colour vision, and optic disc assessment were also reported; however, no natural history data was available for these biomarkers.

In 2008, Cohn et al. [16] reported the natural history and genotyping in an Australian cohort of 158 DOA patients from 11 families. Only right eyes were included, and 69 patients had follow‐up data available, either from prospective review or retrospective records. Visual acuity findings were reported as best corrected Snellen visual acuity from the right eye. They found no statistical difference between initial and final visual acuity (initial: mean 6/18, median 6/24, range 6/4.8‐HM, final: mean 6/18, median 6/36, range 6/4‐HM) across the cohort. They also found 7/69 patients improved by > 2 lines, 43/69 were unchanged, 13/69 worsened by two lines, and 6/69 worsened by > 2 lines. Older patients (> 40 years) were found to have worse mean visual acuity than those aged ≤ 40 years (6/23.3 vs. 6/12.9). Because of the categorical reporting of visual acuity change, it was not possible to calculate rates of change from this study. Thirty‐two patients also had serial optic disc photos which showed no objective change over the follow‐up period (average 6.7 years).

A UK‐based series by Yu‐Wai‐Man et al. [4] in 2010 investigated the prevalence of *OPA1* and *OPA3* mutations in patients with DOA phenotype in Northern England. Forty‐five *OPA1*‐confirmed patients with DOA phenotype were identified. The mean age of onset of visual symptoms was 7 years (SD = 3.9 years), with 80% symptomatic by 10 years of age. The mean visual acuity at last follow‐up was 1.02 LogMAR in genetically confirmed cases, and in 80% the visual acuity was symmetrical between eyes. Visual outcome in patients with missense *OPA1* mutations (mean 1.83 LogMAR) was worse than those with nonsense, deletions or splice site mutations (mean 0.93 LogMAR). Visual outcomes were also worse in patients older than 40 years. Longitudinal data was reported in 43 patients using retrospective chart review with mean follow‐up of 18 years (SD = 14.7 years). 29/43 patients experienced visual decline during their follow‐up. The mean rate of visual loss was 0.032 LogMAR/year (SD = 0.045), with significant variation between 0.0 and 0.171 LogMAR/year. Most recent visual acuity (mean = 1.06, SD = 0.67) was significantly worse than baseline vision (mean = 0.68, SD = 0.53). The mean age at final review was not directly reported for the *OPA1* group with longitudinal data. Fundal examination was also commented upon, with all patients having abnormal optic nerve head appearance; however, no longitudinal data was available.

A year later in 2011, Yu‐Wai‐Man et al. [17] released a similar study with North American data, again investigating the yield of *OPA1* and *OPA3* screening in patients with DOA phenotype. Thirty‐two *OPA1* patients were identified, with a mean age of onset of 10.3 years and 90% becoming symptomatic before 20 years of age. The mean age at final review was 33.1 years (SD = 17.9 years). Longitudinal data was available in 24 patients, with a mean follow‐up of 8.5 years (SD = 9.0 years). 13/24 patients experienced worsening of visual acuity. The rate of visual loss was 0.070 LogMAR/year (SD = 0.098 LogMAR/year), and the mean visual acuity at last follow‐up (0.66 LogMAR) was significantly worse than baseline visual acuity (0.45 LogMAR). The authors highlighted the significant variation in rates of visual loss (0.01–0.40 LogMAR/year), although they did not find any association between visual outcomes and *OPA1* genotypes. Other clinical findings were noted at a single time point, including colour vision, visual fields and optic nerve head appearance.

The first cohort study investigating the effects of idebenone in *OPA1* DOA patients was published in 2020 by Romagnoli et al. [8] Change in visual acuity in the better seeing eye was used as the primary outcome measure, and a change of > 0.1 LogMAR (five letters) was considered a significant change. In the 37 untreated *OPA1* patients, mean follow‐up was 4.2 years (SD = 2.3 years). At baseline, mean best‐corrected visual acuity was 0.7 LogMAR and 0.8 LogMAR in the better and worse seeing eyes, respectively. Median visual acuity at baseline was 0.52 (Q1–Q3 = 0.2–0.9), and at last follow‐up was 0.52 (Q1–Q3 = 0.3–1.0). Median visual acuity change was 0.00 (Q1–Q3 = −0.04–0.13). Paired longitudinal data was not reported. In this study, idebenone was found to stabilise visual acuity change in *OPA1* DOA patients.

Most recently, Arruti et al. [18] published a retrospective case series of paediatric DOA patients with confirmed *OPA1* mutations. The focus of their study was on careful mapping of genotype–phenotype correlations. Eleven children were included over a 4‐year period. Mean visual acuity at baseline was 0.40 LogMAR and 0.44 LogMAR in the right and left eyes, respectively. This was unchanged at last follow‐up. Individual visual acuity and follow‐up periods were not directly reported.

### 3.1 Meta‐Analysis

**FIGURE 2:**
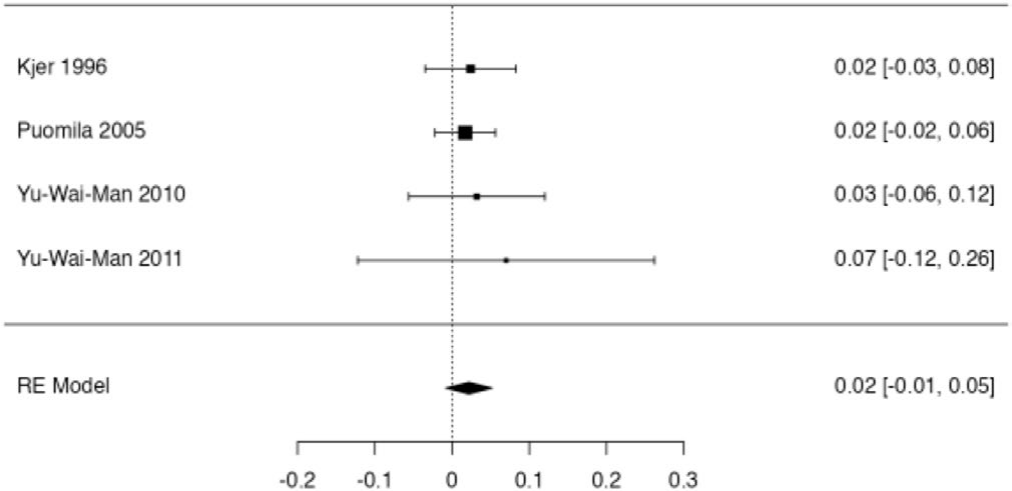
Forrest plot of included studies (left and middle columns), with right column showing the individual effect size (change in LogMAR/year) for each study (± 95% confidence interval). The pooled effect size is seen at the bottom of the figure, labelled RE (Random Effects) model.

A total of *k* = 4 studies were included in the meta‐analysis [4, 14, 15, 17]. These were included as they reported paired data with individual rates of visual change (or paired data was able to be deduced post hoc from their reports). The remaining studies reported global cohort statistics and thus were not included [8, 16, 18]. The observed mean effect size ranged from 0.017 to 0.070 change in LogMAR/year (0.02 = 1 letter on ETDRS chart). The estimated effect size based on the random‐effects model was 0.022 LogMAR/year (95% CI: −0.008 to 0.052). This suggests that the change in visual acuity each year is not significantly different to zero (*Z* = 1.4, *p* = 0.155, see Figure 2). In the random‐effects model, there was no significant difference between study variance (τ^2^ = 0), and the estimate was identical to that of a fixed‐effect model. Heterogeneity across studies was also low (*Q* = 0.36).

## 4 Discussion

The traditional DOA phenotype is one of slowly progressive bilateral visual loss with onset during childhood. However, longitudinal data describing this progression is lacking. Since the *OPA1* gene was first mapped in 1994 [19], only seven papers met our inclusion criteria. These papers detail a slow decline in visual acuity; however, all had at least some DOA patients who did not progress during follow‐up. Our meta‐analysis highlights this slow rate of progression. In fact, although the effect estimate of 0.022 LogMAR/year suggests a loss of roughly one letter annually, this effect was not found to be significantly different from zero (*p* = 0.155).

The sample size, even across this systematic review, is small. This may be expected as it is a rare disease; however, the inconsistencies in reporting visual acuity metrics hamper any form of pooled analysis to increase statistical power. Only two studies [4, 17] directly reported the rate of change in visual acuity per year. In two other studies, this was able to be deduced from published graphics [14, 15]. Although the remaining studies [8, 16, 18] did not detail paired data or rates of change in their subjects, two concluded there was no progression based on their results [16, 18]. A more uniform method of reporting visual acuity and other biomarkers in natural history studies would maximise statistical power to detect change, particularly in uncommon conditions.

Even when longitudinal studies have been performed, there is a paucity of alternative biomarkers being assessed. We did not identify any studies with natural history data for other common biomarkers such as OCT or visual fields. Cohn et al. [16] followed disc appearance in their cohort; however, this data was not expanded upon other than to say there was no difference. It should be noted that many cross‐sectional studies exist looking at other biomarkers including OCT [20, 21, 22, 23], visual field [21, 23, 24], colour vision [24, 25, 26], electrophysiology [27, 28] and optic disc assessment [24, 25, 29] in DOA patients. Although some regression estimates are possible from these studies, this is less accurate and more generalised than paired data and may overlook important phenotypic variations. This is particularly pertinent given the marked heterogeneity of genotype and phenotype seen in DOA cohorts.

When comparing the natural history of visual loss in *m.11778G>A* Leber Hereditary Optic Neuropathy [30, 31], 15 studies were identified and included 695 LHON patients. Compared to DOA, LHON patients typically experience more rapid visual loss, which makes detecting treatment effect potentially easier. However, complicating factors include highly variable progression rates (e.g., whether at nadir vs. at the time of visual loss), the more severe vision loss contributing to a floor effect, and that up to 20% may experience some spontaneous visual recovery [30]. The authors identified similar inconsistencies in how visual acuity was measured and reported and highlighted the need for further robust prospective studies in genetically homogenous LHONs, a conclusion which can be applied to DOA patients also. Despite this, the promising results for lenadogene nolparvovec (rAAV2/2‐ND4) in *m.11778* LHON patients encourage further investigation into genetically targeted therapies for both of these inherited optic neuropathies.

In this systematic review of DOA studies, the pooled rate of visual acuity change is not different from zero. This suggests that, for a similar sample size, it may be relatively more difficult to prove the efficacy of new treatments using best‐corrected visual acuity as the endpoint. Put another way, if the initial goal of new therapies is to stabilise vision, then there will likely be a limited detectable difference in BCVA between the intervention and control groups. Ultimately, the more a new treatment is able to improve vision rather than merely stabilise it, the more tenable the use of BCVA becomes as a primary trial endpoint. Interestingly, the final study [8] of Romagnoli et al. was the only interventional study included in this review, which found a significant stabilisation or improvement in idebenone‐treated DOA patients compared to the untreated DOA group. This conclusion was based on proportions of patients who stabilised or improved BCVA, rather than paired rates of visual decline. Despite not controlling for mutation type, this method may offer a more accessible statistical approach for future trials. It may also be possible to increase the sample size to overcome a small effect size; however, this is a rare disease. A recent review found that for conditions with a prevalence of 1–9/100 000 (such as DOA), the fitted mean sample size of Phase 2 and 3 studies was 33.8 (95% CI: 22.1–51.7) and 75.3 (95% CI: 48.2–117.6) respectively, much lower than trials of more prevalent diseases [10]. An additional option would be to identify subgroups of DOA patients, potentially based on genotype, who are known to progress more rapidly or who are more likely to respond to an intervention.

Ultimately, future trials of DOA therapies pose several challenges to being able to detect meaningful change in visual acuity within a practical study timeline. The use of BCVA as the primary outcome measure becomes problematic when using traditional sample sizes and study durations and risks failing to detect meaningful change for patients. This warrants further longitudinal exploration of alternative biomarkers for use in clinical trials of DOA therapies. It is interesting to speculate about which biomarkers may be useful to investigate in these longitudinal studies. The use of low‐contrast or low‐luminance visual acuity may show greater dynamic range in early disease, as has been shown in other conditions such as multiple sclerosis [32], glaucoma [33] and choroideraemia [34]. OCT is a well‐established structural marker of disease [21, 23]. However, the structural deficits seen in DOA may be complete early in a patient's life [22] and it remains unclear whether rates of nerve fibre layer or ganglion cell layer losses would be detectable within a clinical trial timeframe measured against age‐related changes seen in controls [20]. Visual field testing provides additional functional data but is highly dependent on patient performance and fixation among other factors. Established protocols which assess the central 10° from fixation may not adequately assess early centrocaecal scotomas emanating from the natural blind spot (15°), while more comprehensive 30° testing perhaps unnecessarily involves more peripheral field. Custom field maps may allow better assessment of centrocaecal scotomas, while microperimetry allows for additional measures of fixation [21]. Particularly when combined with volumetric assessment, these approaches warrant further investigation in this population [35]. Electrophysiology markers such as the photopic negative response offer additional insight into ganglion cell function in DOA [27, 36]. However, it remains to be established how this compares to the pattern visual evoked potential or pattern electroretinogram in the clinical trial setting. Newer technology such as flavoprotein fluorescence allows for mitochondrial‐specific imaging, while DARC allows real‐time visualisation of retinal cell apoptosis. Both show promise as targeted biomarkers and have been utilised in glaucoma [37, 38, 39] and age‐related macular degeneration [40] cohorts; however, their utility in DOA patients remains to be seen. Given the clinical course of DOA, it is unlikely that any of these tests will show a large amplitude of change over time. Thus, the test–retest variability and ability for reproducible measurements in the same patient with tight confidence intervals becomes increasingly important to be able to detect meaningful change in the clinical trial setting and warrants further natural history data for each prospective biomarker.

## 5 Conclusion

In this review we attempted to characterise the rate of longitudinal change in visual biomarkers in DOA patients. The very slow rate of progression in DOA patients, coupled with the paucity of available longitudinal data on other biomarkers, raises important considerations for future study design and evaluation of emerging therapies. This highlights the need for further natural history studies and a better understanding of phenotypic and genotypic heterogeneity in DOA patients.

## Conflicts of Interest

The authors declare no conflicts of interest.

## Data Availability

The data that support the findings of this study are available from the corresponding author upon reasonable request.

## Appendix A MEDLINE Search Strategy

1. Dominant Optic Atrophy.mp, or Optic Atrophy, Autosomal Dominant/
2. DOA.mp
3. ADOA.mp
4. Dominant Optic Atrophy Plus.mp
5. DOA Plus.mp
6. DOA+.mp
7. Kjer.mp
8. OPA1
9. or/1–8
10. Biomarker*.mp, or Biomarkers/
11. Outcome*.mp
12. Visual outcome*.mp
13. Visual Acuity.mp, or Visual Acuity/Perimetry.mp
14. Visual Field.mp, or Visual Field Tests/
15. Electrophysiology.mp, or Electrophysiology/
16. Electroretinography/
17. VEP.mp, or Visual Evoked Potential*.mp, or Evoked Potentials, Visual/
18. Colo?r vision.mp, or Colour Perception Tests/, or Colour Perception/
19. Patient reported outcome measures.mp or Patient Reported Outcome Measures/
20. or/10–20
21. Limit human
22. 9 and 21 and 22

This strategy was adapted for the other databases, as listed in the text. ‘.mp’ denotes keyword search, while ‘/’ denotes subject headings.
